# Microbial Uptake, Toxicity, and Fate of Biofabricated ZnS:Mn Nanocrystals

**DOI:** 10.1371/journal.pone.0124916

**Published:** 2015-04-22

**Authors:** Brian J. F. Swift, Franҫois Baneyx

**Affiliations:** Department of Chemical Engineering, University of Washington, Seattle, Washington, United States of America; Arizona State University, UNITED STATES

## Abstract

Despite their importance in nano-environmental health and safety, interactions between engineered nanomaterials and microbial life remain poorly characterized. Here, we used the model organism *E*. *coli* to study the penetration requirements, subcellular localization, induction of stress responses, and long-term fate of luminescent Mn-doped ZnS nanocrystals fabricated under “green” processing conditions with a minimized ZnS-binding protein. We find that such protein-coated quantum dots (QDs) are unable to penetrate the envelope of unmodified *E*. *coli* but readily translocate to the cytoplasm of cells that have been made competent by chemical treatment. The process is dose-dependent and reminiscent of bacterial transformation. Cells that have internalized up to 0.5 μg/mL of nanocrystals do not experience a significant activation of the unfolded protein or SOS responses but undergo oxidative stress when exposed to high QD doses (2.5 μg/mL). Finally, although they are stable in quiescent cells over temperatures ranging from 4 to 42°C, internalized QDs are rapidly diluted by cell division in a process that does not involve TolC-dependent efflux. Taken together, our results suggest that biomimetic QDs based on low toxicity inorganic cores capped by a protein shell are unlikely to cause significant damage to the microbial ecosystem.

## Introduction

Quantum dots (QDs) are semiconductor nanocrystals that are gaining in popularity over organic fluorophores in applications ranging from bioimaging[1] and analytical assays[2] to electronic displays,[3] solid-state lighting[4] and photovoltaics.[5] QDs commonly consist of a CdSe, CdTe, ZnSe, or PbSe core coated with a ZnS shell to enhance stability and optical properties. In some cases, the shell is further functionalized with thiols or amphiphilic polymers to make the nanocrystals soluble in aqueous solvents and to allow for biomolecule conjugation.[6–8] As QDs become more prevalent in consumer products that will be used, reused, recycled, and landfilled, concerns have been rising about their impact on humans and the environment.[9]

Studies conducted with cultured eukaryotic cells have revealed that QDs can exert cytotoxic effects through a variety of mechanisms. These include leaching of toxic heavy metals from the inorganic core, (photo)generation of reactive oxygen species that induce oxidative stress, and direct or indirect damage to genomic DNA and biological membranes.[6,10–13] Parameters such as size, shape, composition, and surface coating(s) can all impact cytotoxicity outcomes and do so in a mechanism-specific (and cell-specific) [14] manner. For instance, while ZnS shells or polymer coatings can reduce the cellular toxicity of CdSe nanocrystals,[11] they do little to prevent photo-induced DNA damage.[15] Similar observations have been made in animals, where the situation is further complicated by the route of exposure and where long-term retention in the liver, spleen, kidney, and lymph nodes is of primary concern.[10,16,17]

There is considerably less information on how QDs interact with prokaryotes although these abundant microorganisms will be first to come into contact with engineered nanomaterials that find their way into the environment. Furthermore, most studies conducted to date have focused on cadmium-based QDs (CdS, CdSe and CdTe cores) produced with different synthesis schemes and coatings, and used at different doses with a variety of strains and culture conditions.[18–20]

To answer a growing demand for the production of functional nanomaterials through environmentally friendly processes, we previously described a set of “designer” proteins that support the low-temperature and aqueous fabrication of undoped and transition metal-doped ZnS QDs to which antibodies can be conjugated by simple mixing.[21–23] Because zinc is not as toxic as cadmium,[24] these particles should exhibit low cellular toxicity upon core dissolution, and because they are capped by proteins as part of the manufacturing process, their shell is already biologically-relevant.

Here, we used *E*. *coli* as a model organism to investigate the penetration requirements, subcellular localization, induction of stress responses, and long-term fate of luminescent ZnS:Mn nanocrystals fabricated with a minimized designer protein.[23] Our results suggest that such protein-coated fluorophores are environmentally benign because their uptake requires membrane destabilization, they only induce oxidative stress at high doses, and they are rapidly diluted by cell division.

## Results and Discussion

### Uptake by *Escherichia coli* requires membrane destabilization

**Fig 1:**
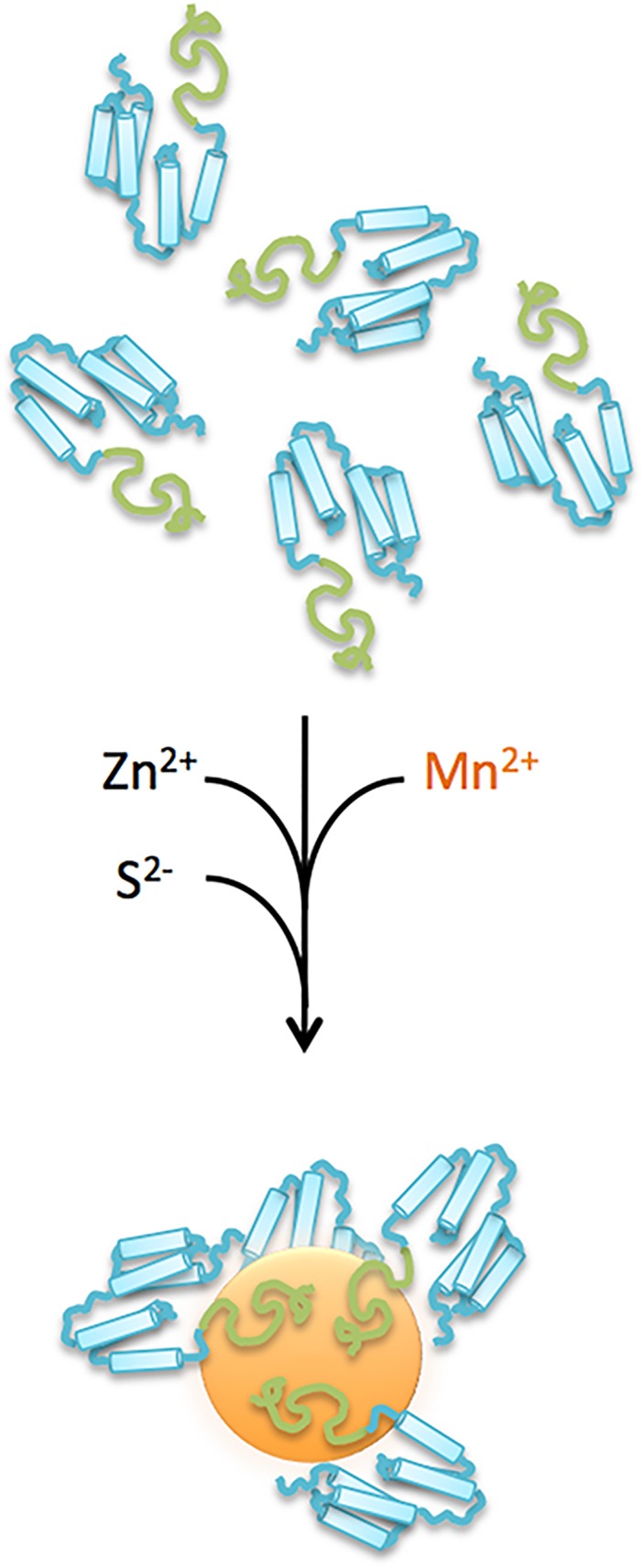
Schematic illustration of the protein-aided QDs synthesis process. The BB-CT43 protein consists of a tandem repeat of the IgG binding domain from *S*. *aureus* protein A (blue) followed by the CT43 zinc sulfide binding peptide (green). Purified BB-CT43 is mixed at a concentration of 5 μM with a solution of zinc and manganese acetate and formation of Mn-doped ZnS particles is induced by dropwise addition of sodium sulfite as described.[22] The ZnS:Mn nanocrystals are capped by BB-CT43 before their growth exceeds about 4 nm and are ready to use after 4 days of aging at 37°C.

We recently reported that BB-CT43, a minimized designer protein consisting of a linear ZnS binding peptide (CT43) fused to an antibody-binding domain derived from *S*. *aureus* Protein A (BB) is suitable for the one-pot synthesis of ZnS:Mn QDs.[23] These luminescent nanocrystals are produced when the designer protein caps the growth of the inorganic core at about 4 nm,[22] a process that is schematically illustrated in Fig 1. With their protein shell, the particles have an overall hydrodynamic diameter of 9.5 ± 2 nm and a zeta potential of -16.5 ± 6 mV. They exhibit a strong emission peak at 590 nm under UV illumination, can be decorated with antibodies by simple mixing and are stable for months without aggregation or degradation of optical properties.[23] Unlike traditional QDs, these fluorophores are manufactured using mild aqueous conditions, do not contain highly toxic heavy metals such as cadmium, and sport a built-in protein shell coat. Thus, they should have a minimal impact on microbial life and the environment.

As a first test of this hypothesis, we studied the uptake of BB-CT43-stabilized QDs by *Escherichia coli*, a well-studied gram-negative organism whose envelope consists of a 5 nm-thick phospholipid bilayer inner membrane, a 12 nm-thick interstitial space known as the periplasm, a 1 to 2 nm-thick peptidoglycan layer, and a 13 nm-thick, negatively charged outer membrane composed of a lipopolysaccharide outer leaflet and a phospholipid inner leaflet.[25]

Like all prokaryotes, *E*. *coli* lacks the endocytosis pathways responsible for nanoparticle uptake by eukaryotes. In addition, bacterial porins, which allow free diffusion of small molecules across prokaryotic membranes through 1–2 nm pores,[26] should be too small to allow even the smallest QDs to enter the cell. Nevertheless, Hirschey and coworkers reported that CdSe/CdS QDs stabilized by citrate, isocitrate, succinate, or malate readily penetrate *E*. *coli* when their inorganic core is smaller than 6 nm. By contrast, Wenhua *et al*. found that the uptake of mercaptoacetic acid-stabilized QDs with 3 to 4 nm CdSe/CdS cores require chemical destabilization of the outer membrane,[27] while Nadeau and coworkers reported that internalization of adenine-coated CdSe QDs strictly depends on photo-induced membrane damage and purine metabolism.[18]

**Fig 2:**
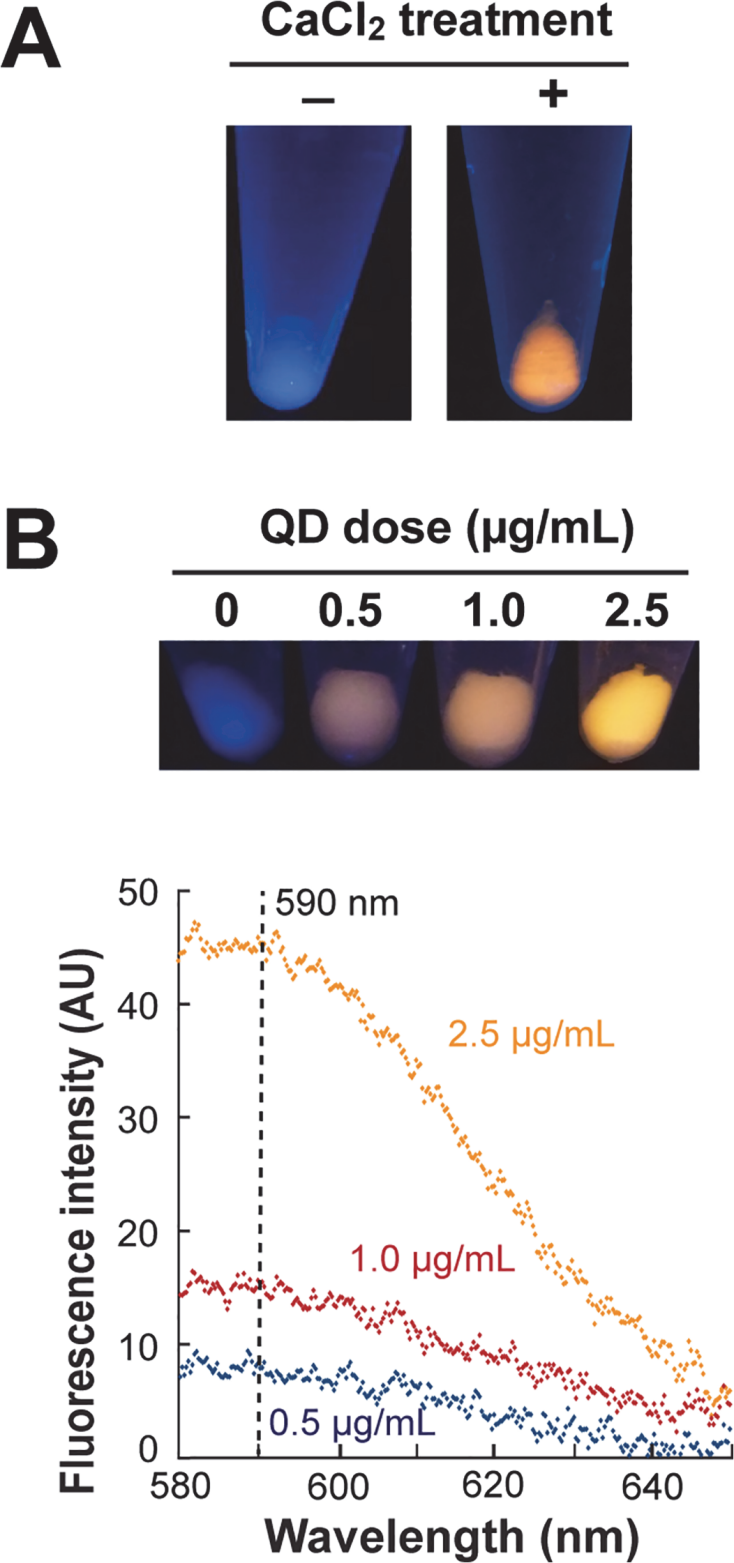
Membrane destabilization is required for QD uptake. (A) AB734 cells made competent (+) or not (-) by treatment with CaCl_2_ on ice were incubated with 0.5 μg/mL of BB-CT43-stabilized ZnS:Mn nanocrystals for 2h at room temperature. Cells were washed twice, centrifuged, and pellets were photographed under UV illumination. (B) Competent cells were incubated with the indicated concentration of QDs and photographed as above. Fluorescence emission spectra of cell suspensions were recorded following excitation at 280 nm.

To determine whether BB-CT43-stabilized nanocrystals would be uptaken by unmodified *E*. *coli*, we incubated 0.5 μg/mL of nanoparticles with mid-exponential phase *E*. *coli* cells for 2h at room temperature. Cells were washed to remove nonspecifically bound particles, pelleted by centrifugation and exposed to UV light. Under these conditions, there was no detectable nanocrystal uptake (Fig 2A). However, in agreement with the results of Wenhua and coworkers,[27] fluorescent material colocalized with sedimented cells if they were first made chemically competent by incubation with 100 mM CaCl_2_ at low temperature. This treatment transiently affects the integrity of the outer membrane and is routinely used for introducing naked DNA into cells, although the precise mechanisms at play remain unknown.

Not unexpectedly, uptake was dose-dependent and we observed a linear increase in cell fluorescence at 590 nm (the QD emission maximum) when the particle concentration was increased from 0.5 to 2.5 μg/mL (Fig 2B). We conclude that chemical disruption of the outer membrane is required for the uptake of QDs coated with protein shells and whose inorganic cores are less photo reactive than CdSe/CdS.

### Internalized QDs localize to the cytoplasm

**Fig 3:**
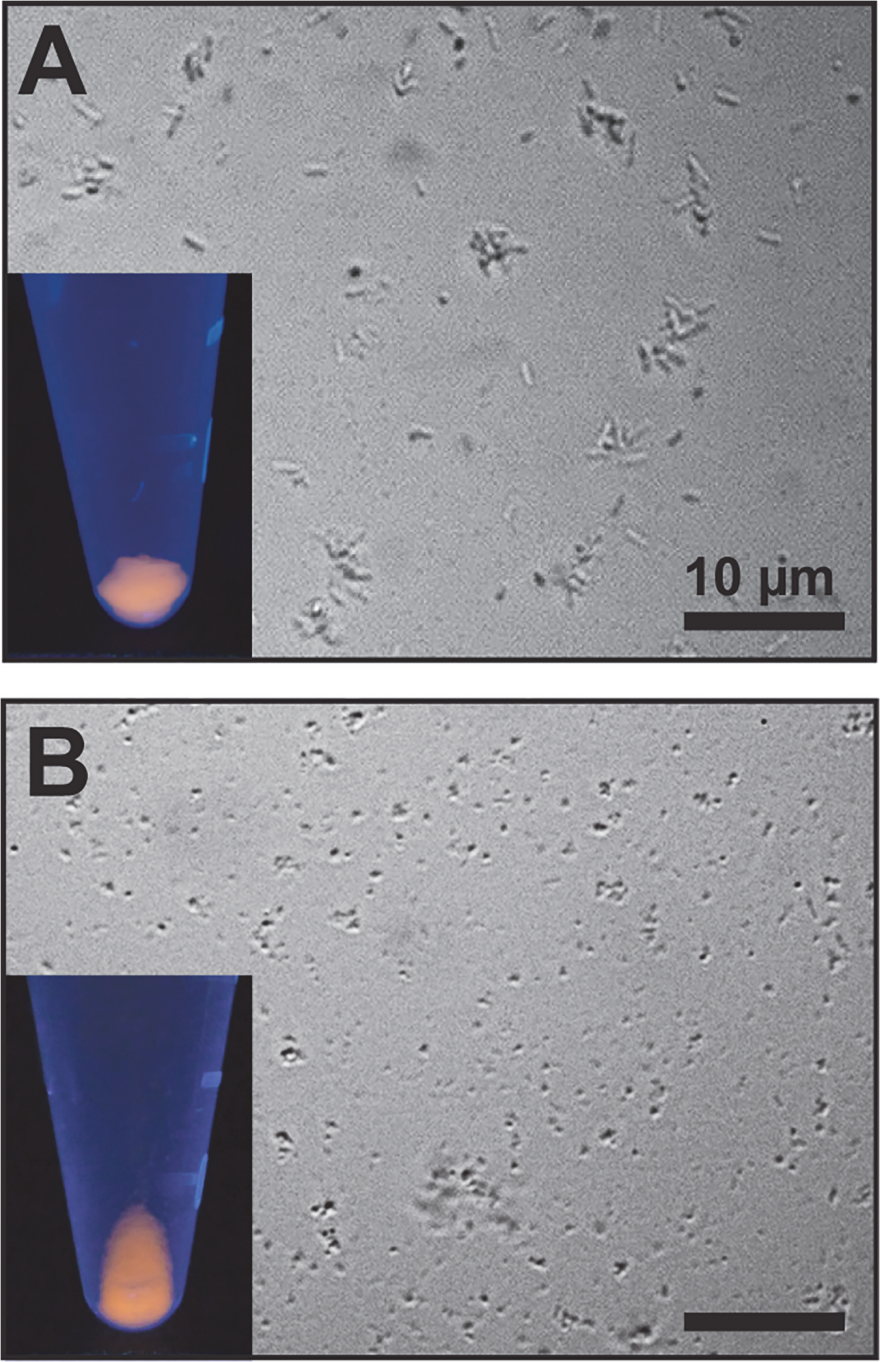
Spheroplasts remain fluorescent. Competent AB734 cells were incubated with 0.5 μg/mL of BB-CT43-stabilized QDs, washed and imaged on an optical microscope without further treatment (A) or following spheroplasting (B). Insets show the appearance of pelleted cells or spheroplasts upon UV illumination.

To confirm that the nanocrystals were not simply adsorbed to the surface of the outer membrane, competent cells incubated with 0.5 μg/mL of BB-CT43-stabilized QDs as above were subjected to spheroplasting (Fig 3). This procedure strips *E*. *coli* of its outer membrane and peptidoglycan layer, cause release of periplasmic contents in the surrounding medium, and leads to loss of rod shape and the formation of spherical vesicles bounded by the inner membrane.[28] Fig 3B shows that spheroplasts remained fluorescent, indicating that the QDs were either associated with the inner membrane or had translocated to the cytoplasm.

Because confocal microscopy does not allow one to unambiguously distinguish between these two possibilities, we took advantage of the fact that the emission spectrum of UV-excited ZnS:Mn nanocrystals overlaps the absorption spectrum of the fluorescent protein mCherry.[29] Thus, Forster Resonance Energy Transfer (FRET) should occur between the two fluorophores if they co-localize to the same cellular compartment and are separated by distances smaller than 10 nm.

**Fig 4:**
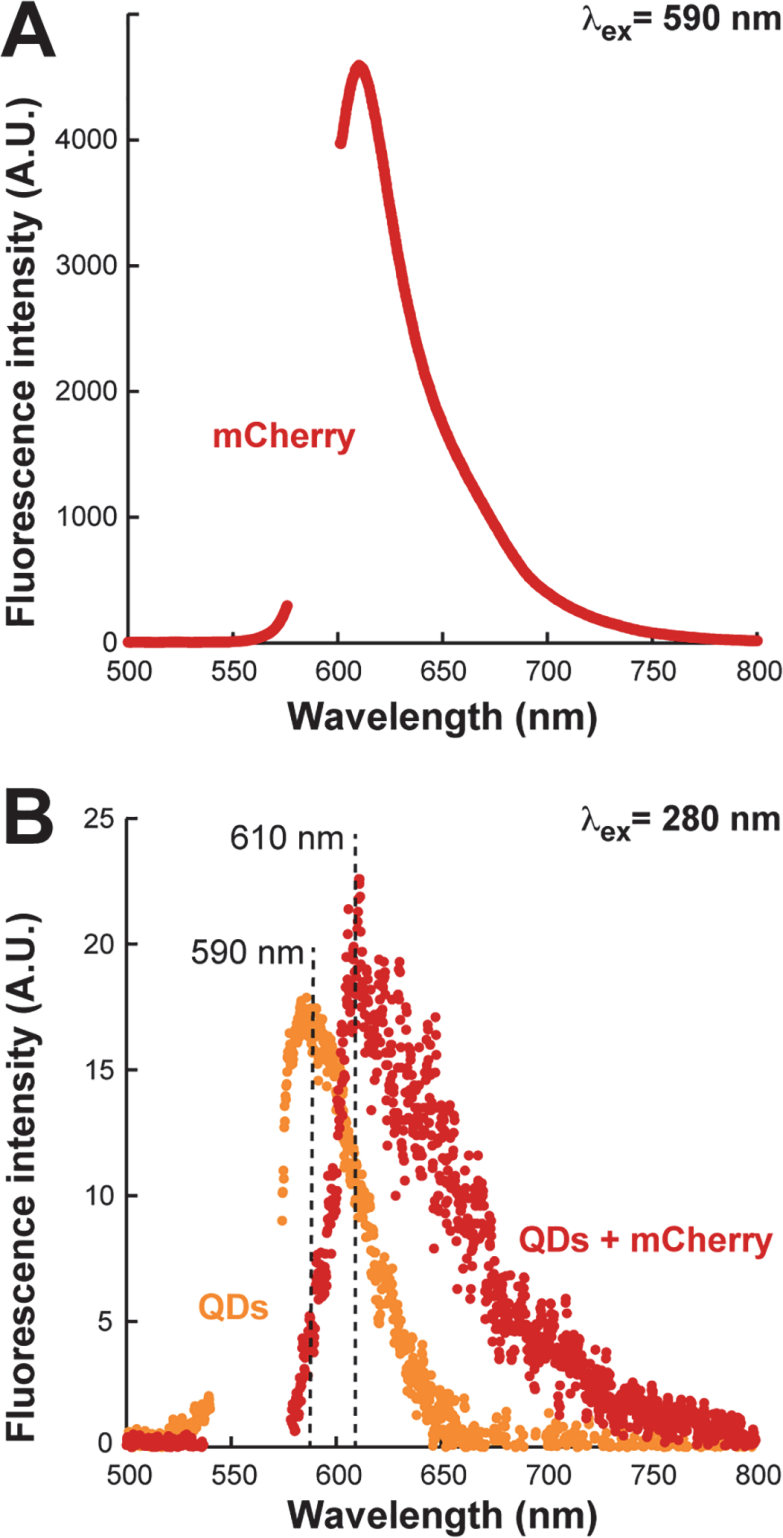
FRET confirms cytoplasmic localization. (A) Fluorescence emission spectrum of mCherry-producing cells upon excitation at 590 nm. (B) Fluorescence emission spectra of AB734 cells that had internalized 0.5 μg/mL of BB-CT43-stabilized QDs following excitation at 280 nm without (orange) or with (red) concomitant production of cytoplasmic mCherry. See text for details.

To test this idea, we first recorded the fluorescence emission spectra of competent cells that had taken up QDs or had been exposed to buffer alone following excitation at 280 nm. Subtraction of the two spectra eliminated the contribution of background fluorescence and revealed a weak but clear peak centered at 590 nm and corresponding to ZnS:Mn emission (Fig 4, orange). Next, we introduced a plasmid expressing mCherry at high level in the cytoplasm of *E*. *coli* and confirmed that the emission spectrum of these cells exhibited the characteristic shape and 610 nm emission maximum of mCherry upon excitation at 590nm (Fig 4, inset).[29] Finally, we made competent mCherry-producing cells, exposed them to QDs or buffer, and collected fluorescence emission data following excitation at 280 nm. The subtracted spectrum (Fig 4, red) shows that the QD-associated peak at 590 nm peak completely disappeared to the profit of a 610 nm peak corresponding to mCherry emission. We conclude that nonradiative energy transfer occurs between QDs (donor) and mCherry (acceptor) and therefore that both species are located within a few nanometers of each another in the cytoplasm.

How QDs (or plasmid DNA for the matter) translocate across the peptidoglycan layer, periplasm and inner membrane to reach the cytoplasm remains unclear. One possible explanation is that they rely on the transient and CaCl_2_-induced opening of sites where the outer and inner membranes come into intimate contact. Such adhesion zones, known as Bayer’s patches, were identified microscopically over 40 years ago,[30] but their existence has remained controversial in spite of supporting biochemical evidence.[31]

### High doses of ZnS:Mn nanocrystals are required to induce an oxidative stress responses

We next investigated how the presence of BB-CT43-stabilized nanocrystals in the cytoplasm would impact cell physiology. Prokaryotes have evolved complex and redundant mechanisms to survive exposure to environmental stresses. Many of these processes rely on increasing the synthesis of protective proteins (e.g., molecular chaperones, proteases, DNA repair enzymes and reductases) through upregulation events that are often controlled at the transcriptional level. Previously, we described *E*. *coli* cells harboring single-copy gene fusions between the stress-inducible *ibp* or *sulA* promoters and the *lacZ* gene (which encodes β-galactosidase).[32,33] These strains report on the amount of stress experienced by the cell as a result of cytoplasmic protein misfolding (*ibp*::*lacZ* fusion) or DNA damage (*sulA*::*lacZ* fusion) by producing the easily assayed enzyme, β-galactosidase. Because QD cytotoxicity has repeatedly been correlated with oxidative damage,[18,34–37] we constructed an additional isogenic strain bearing a single-copy gene fusion between the oxidative stress responsive promoter of the major *E*. *coli* catalase (the OxyR-regulated *katG* gene product)[38] and *lacZ*.

**Fig 5:**
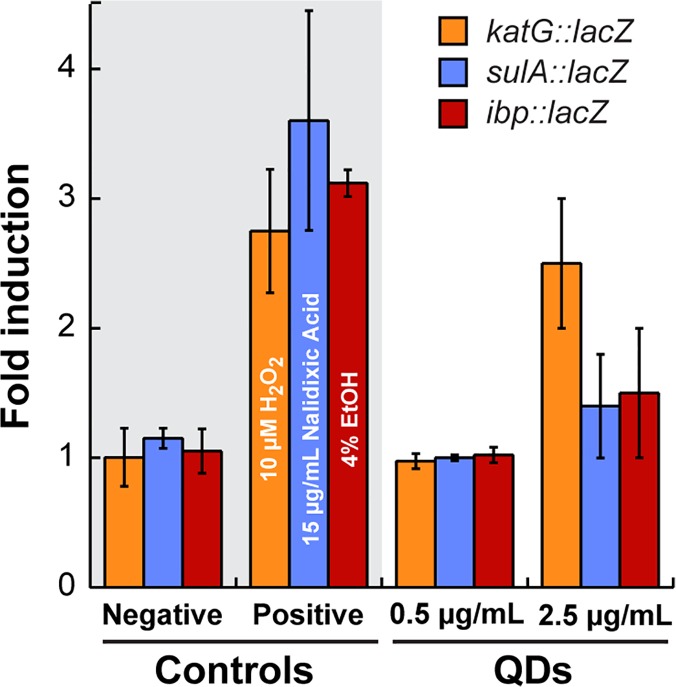
Induction of stress responses. AB734 cells bearing single copy chromosomal fusions between the *lacZ* reporter gene and promoters induced by oxidative stress (*katG*), DNA damage (*sulA*), or cytoplasmic protein misfolding (*ibp*) were made competent and exposed to buffer (negative control) or the indicated chemical stressors. β-galacosidase activities were determined after 3h at 37°C and the magnitude of promoter induction was calculated by assigning a value of unity to the mean of negative controls. The β-galactosidase activity in cultures that had internalized BB-CT43-stabilized QDs supplied at 0.5 or 2.5 μg/mL was determined in a similar fashion. Error bars correspond to independent triplicate experiments.

The functionality of the reporter panel was first confirmed using hydrogen peroxide, nalidixic acid and ethanol at concentrations known to cause extensive oxidative stress, DNA damage, or protein misfolding, respectively.[32,33] These chemicals caused an about 3-fold induction of the corresponding promoters (Fig 5, positive controls). Next, the three strains were made chemically competent, exposed to QDs, and cultures were assayed for β-galactosidase activity after 3h. While there was no detectable induction of any of the stress promoters when QDs were supplied at the concentration used in all above experiments (0.5 μg/mL), addition of 2.5 μg/mL nanocrystals was as effective as the use of 10 μM H_2_O_2_ in inducing the *katG* promoter. Of note, however, there was no statistically significant activation of either the *ibp* or *sulA* promoter under the same conditions (Fig 5). While the dependency of toxicity on QD dose is not particularly surprising,[11] our results indicate that it takes highly concentrated solutions of nanocrystals to fully induce the bacterial oxidative stress response and that BB-CT43-stabilized QDs do not cause appreciable protein misfolding or DNA damage under the same conditions.

### QD fluorescence is rapidly lost in growing cells

The persistence of toxicants in the environment can lead to their long-range transport and bioaccumulation at toxic doses in animal and human tissues. To gain information on the *in vivo* stability of BB-CT43-stabilized QDs, we first incubated cells that had internalized nanocrystals in phosphate buffered saline (PBS) for 24h at temperatures ranging from 4 to 42°C. There was no significant change in the fluorescence of cell pellets indicating that protein-capped nanocrystals are stable for extended periods of time in quiescent cells exposed to a physiologically relevant range of temperatures (S1 Fig).

**Fig 6:**
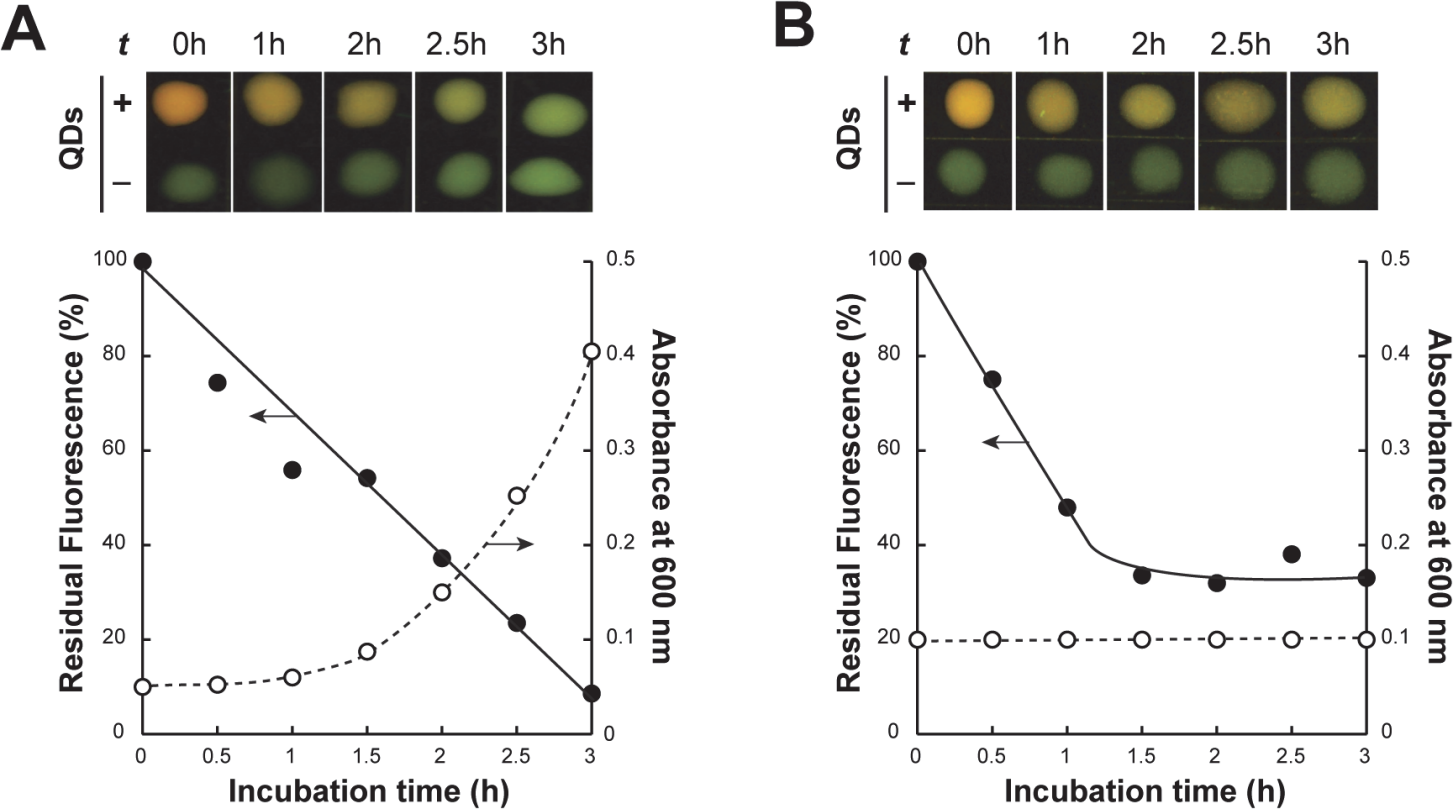
Active cell growth leads to complete elimination of QD fluorescence. The fluorescence (closed circles) and turbidity (open circles) of AB734 cells that had internalized 0.5 μg/mL of BB-CT43-stabilized QDs was measured in the absence (A) or presence (B) of 50 μg/mL of the translational inhibitor kanamycin. Photographs show the fluorescence of cell samples (50 μL) from cultures supplied (+) or not (-) with QDs after the indicated incubation times at 37°C in LB medium. Fluorescence was quantified as described in Materials and Methods.

To determine if growth or metabolic activity would influence this outcome, QD-loaded cells were taken in LB medium or PBS and incubated at 37°C, the optimum growth temperature for *E*. *coli*. While non-growing cells held in PBS did not lose their initial fluorescence (S2 Fig), we observed a linear decrease in fluorescence over time and nearly complete disappearance of the signal after 3h of cultivation in LB medium (Fig 6A, closed symbols). Because cells experienced a 1h lag phase and exponential growth only started about 2h after transfer to LB (Fig 6A, open symbols), the nearly 50% loss of fluorescence that occurs over the first 1.5h of cultivation cannot be attributed to QD dilution by cell division. Indeed, when the experiment was repeated in the presence of the translational inhibitor kanamycin, we observed a similar fluorescence loss over the first 1.5h but, remarkably, no further decrease thereafter (Fig 6B). Thus, although *de novo* protein synthesis and/or cell growth are not implicated in initial signal loss, they are necessary for complete elimination of QD fluorescence.

There are several possible explanations for the initial fluorescence loss: dissolution or extrusion of about 50% of the internalized nanocrystals or substitution of the BB-CT43 shell by host species that change the QD optical properties. We do not believe that chemical dissolution of the nanocrystals is a likely mechanism since it would be unlikely to abruptly stop in kanamycin-treated (Fig 6B) or quiescent cultures (S1 and S2 Figs).

To directly test the possibility that active extrusion was involved, we repeated the experiment of Fig 6A in isogenic cells containing or lacking TolC, a trans-periplasmic protein that functions as an exit duct for the expulsion of a wide variety of small drugs and proteins from the cytoplasm to the growth medium.[39] The lack of significant difference in the kinetics and extent of fluorescence loss in *tolC* ^+^ and *tolC* ruled out the involvement of TolC-dependent QD export (S3 Fig).

While we cannot rule out extrusion through other systems, we favor a mechanism in which endogenous species replace at least some of the bound BB-CT43 at the ZnS:Mn surface and cause a decrease in emission intensity through fluorescence quenching. Such quenching phenomena have previously been described and exploited for ZnS:Mn QDs.[40] This explanation is consistent with our previous finding that ZnS:Mn nanocrystals fabricated with BB-TrxA::CT43 have about 30% lower emission intensity than those synthesized with BB-CT43 owing to fluorescence quenching by the TrxA domain.[23] It is also in agreement with the fact that the fluorescence of kanamycin-treated cultures reaches a plateau after 1.5h (Fig 6B), a time that is presumably needed to modify the surface of all internalized nanocrystals. Why samples taken in PBS do not experience a similar initial decrease in fluorescence (S2 Fig) remains unclear but the process appears to require metabolic activity. Irrespective of the precise mechanism of initial fluorescence loss, the data of Fig 6A shows that the QD signal is rapidly lost in actively growing cultures due to dilution by cell division.

## Conclusions

We have shown here that protein-coated ZnS:Mn nanocrystals can translocate in a dose-dependent manner to the cytoplasm of *E*. *coli*. The process requires transient destabilization of the cell outer membrane and is reminiscent of bacterial transformation. Once in the cytoplasm, biofabricated QDs do not cause a significant induction of the unfolded protein or SOS responses. However, they lead to oxidative stress when supplied at very high concentrations (2.5 μg/mL). Although internalized QDs are stable over a broad range of temperature in quiescent cells, they are rapidly diluted in dividing cells. Taken together, our results suggest that biomimetic fluorophores designed with low toxicity cores and biologically-relevant shells are unlikely to cause significant damage to the microbial ecosystem. These design principles may prove useful for the production of other environmentally benign nanomaterials.

## Materials and Methods

### QD uptake by competent cells

AB734, an *E*. *coli* K-12 strain containing a mutation in the *lacZ* gene but otherwise wild type was obtained from the *E*. *coli* Genetic Stock Center. To prepare competent cells, 500 mL cultures were grown in LB medium at 37°C to *A*_600_ ≈ 0.4, and cells were sedimented by centrifugation at 8,000*g* for 8 min and resuspended in 100 mL of 100 mM CaCl_2_ or phosphate buffered saline (PBS;150 mM NaCl, 10 mM Na_2_HPO_4_, 2 mM KH_2_PO_4_). After 30 min incubation on ice and centrifugation at 8,000*g* for 8 min, cells were taken into 12.5 mL of 100 mM CaCl_2_ (or PBS for a non-competent control) and held on ice overnight. Glycerol was added to a 10% (v/v) final concentration and aliquots (200 μL) were stored at -80°C for future use. For uptake experiments, competent or control cells were thawed at room temperature, washed twice with PBS with intervening cycle of centrifugation at 4,000 rpm for 5 min in a microfuge, and resuspended in 900 μL of the same buffer. QDs (approximately 100 μL for a dose of 0.5 μg/mL) were added and the mixture was incubated at room temperature for 2h without shaking. Cells were washed twice with PBS to remove unincorporated QDs.

### QD subcellular localization

Cells that had uptaken QDs were stripped of their outer membrane and peptidoglycan layer by spheroplasting.[28] Briefly, samples prepared as above were resuspended in 200 μL of buffer A (100 mM Tris-HCl, pH 8.0, 0.5 M sucrose, 0.5 mM EDTA), and 10 μL of a 2 mg/mL solution of lysozyme was added, followed by 400 μL buffer A, and 400 μL of ddH_2_O. After 20 min at room temperature, spheroplasts were recovered by centrifugation at 12,800*g* for 30 s and resuspended in 100 mM Tris-HCl, pH 8.0, 0.3 M sucrose, 10 mM MgCl_2_. Samples were visualized on an optical microscope at 50x magnification.

### FRET experiments

AB734 (pmCherry-mut2) cultures were grown to *A*_600_ = 0.4 at 37°C in LB medium supplemented with 50 μg/mL kanamycin. Production of mCherry was induced by addition of 0.2% L-arabinose and cultures were collected after 3 h of growth at 37°C. Cells were made competent by CaCl_2_ and ice treatment as above and stored in 200 μL aliquots. After two wash cycles and resuspension in 900 μL PBS, one sample was incubated for 2 h with 100 μL of BB-CT43-stabilized QDs or the same volume of PBS to serve a control. After 2 wash cycles with PBS, samples were diluted 20-fold in PBS and fluorescence emission spectra were recorded with excitation at 280 nm or 590 nm. Control samples of AB734 cells lacking the pmCherry-mut2 plasmid and incubated or not with QDs were prepared as above and fluorescence spectra were recorded following excitation at 280 nm. The spectra of Fig 4 show QD-free AB734 emission subtracted from QD-treated AB734 emission with excitation at 280 nm (orange), and QD-free mCherry-producing AB734 subtracted from QD-treated mCherry-producing AB734 with excitation at 280 nm (red).

### QD fate

Aliquots (200 μL) of AB734 cells that had uptaken QDs as above were used to inoculate 2 mL of LB media in multiple 15 mL culture tubes supplemented or not with 50 μg/mL of the translational inhibitor kanamycin. Cultures were transferred to 37°C water bath. At the indicated time points, culture absorbance was recorded at 600 nm and samples (2 mL) were subjected to centrifugation at 5,000 rpm for 5 min in a microfuge. Cells were resuspended in 50 μL of PBS, deposited on quartz microscope slide and photographed on a UV table with excitation at 303 nm. Median fluorescence in square areas encompassing about 60% of the droplets and excluding their edges was quantified in the red channel using the histogram function of Adobe Photoshop. Fluorescence loss was quantified by subtracting the fluorescence of control samples from that of QD-loaded cells at the indicated time points.

### Stress responses

Strains ADA110 (AB734 λϕ*ibp*::*lacZ*)) and ADA510 (AB734 λϕ*sulA*::*lacZ*)) have been described previously.[32,33] ADA710 (AB734 λϕ*katG*::*lacZ*)) was constructed by lysogenizing AB734 with a bacteriophage λ derivative bearing the oxidative stress-responsive *katG*::*lacZ* translational fusion and isolated from BGF931 (a kind gift from Dr. Gonzalez-Flecha) through standard protocols.[41] The three strains were made chemically competent by CaCl_2_ treatment and incubated or not with QDs as above. After resuspension in buffer, 200 μL of culture was used to inoculate 5 mL of LB medium. Samples were either exposed to buffer (negative control), known stress response inducers (4% ethanol, 15 μg/mL nalidixic acid, or 10 μM H_2_O_2_) or 0.5 μg/mL or 2.5 μg/mL of BB-CT43-stabilized QDs. After 3h incubation at 37°C, cells were lysed and β-galactosidase activities determined as described. [42]

### Analytical techniques

UV-visible absorption spectra were recorded on a Beckman DU640 spectrophotometer. Fluorescence and phosphorescence emission spectra were recorded using 1 mL of sample on a Hitachi F4500 fluorescence spectrophotometer with excitation at 280 nm and excitation and emission slit widths set at 2.5 nm (fluorescence) or excitation at 316 nm and excitation and emission slit width at 2.5 nm and 10 nm, respectively (phosphorescence). The wavelength region corresponding to the second order diffraction peak of the excitation light was omitted.

## Supporting Information

S1 Fig Quiescent cells that have internalized QDs remain fluorescent over a broad range of temperatures. Competent AB734 cells incubated with 0.5 μg/mL of BB-CT43-stabilized QDs remain fluorescent after 24h of incubation in PBS buffer at temperatures varying from 4 to 42°C.
(PDF)

S2 Fig Quiescent cells that have internalized QDs remain fluorescent over time. Competent AB734 cells incubated with 0.5 μg/mL of BB-CT43-stabilized QDs remain fluorescent after 3h of incubation in PBS buffer at 37°C while they completely lose fluorescence under the same conditions in LB medium.
(PDF)

S3 Fig Impact of a *tolC* null mutant on fluorescence loss. Inactivation of *tolC* has no obvious impact on the loss of fluorescence in AB734 cells experiencing balanced growth in LB medium at 37°C.
(PDF)

## References

[1] Zrazhevskiy P, Sena M, Gao X. Designing multifunctional quantum dots for bioimaging, detection, and drug delivery. Chem Soc Rev. 2010; 39: 4326–4354. 10.1039/b915139g 20697629 PMC3212036

[2] Frigerio C, Ribeiro DS, Rodrigues SS, Abreu VL, Barbosa JA, Prior JA, et al Application of quantum dots as analytical tools in automated chemical analysis: a review. Anal Chim Acta. 2012; 735: 9–22. 10.1016/j.aca.2012.04.042 22713912

[3] Sun Q, Wang A, Li LS, Wang D, Zhu T, Xu J, et al Bright, multicoloured light-emitting diodes based on quantum dots. Nature Photon. 2007; 1: 717–722.

[4] Shirasaki Y, Supran GJ, Bawendi MG, Bulovic V. Emergence of colloidal quantum-dot light-emitting technologies. Nature Photon. 2013; 7: 13–23.

[5] Nozik AJ, Beard MC, Luther JM, Law M, Ellingson RJ, Johnson JC. Semiconductor quantum dots and quantum dot arrays and applications of multiple exciton generation to third-generation photovoltaic solar cells. Chem Rev. 2010; 110: 6873–6890. 10.1021/cr900289f 20945911

[6] Biju V, Itoh T, Anas A, Sujith A, Ishikawa M. Semiconductor quantum dots and metal nanoparticles: syntheses, optical properties and biological applications. Anal Bioanal Chem. 2008; 391: 2469–2495. 10.1007/s00216-008-2185-7 18548237

[7] Jamieson T, Bakhshi R, Petrova D, Pocock R, Imani M, Seifalian AM. Biological applications of quantum dots. Biomaterials. 2007; 28: 4717–4732. 17686516 10.1016/j.biomaterials.2007.07.014

[8] Medintz IL, Uyeda HT, Goldman ER, Mattoussi H. Quantum dot bioconjugates for imaging, labeling and sensing. Nat Mater. 2005; 4: 435–446. 15928695 10.1038/nmat1390

[9] Winnik FM, Maysinger D. Quantum dot cytotoxicity and ways to reduce it. Acc Chem Res. 2013; 46: 672–680. 10.1021/ar3000585 22775328

[10] Bottrill M, Green M. Some aspects of quantum dot toxicity. Chem Commun (Camb). 2011; 47: 7039–7050. 10.1039/c1cc10692a 21475767

[11] Derfus AM, Chan WCW, Bhatia SN. Probing the cytotoxicity of semiconductor quantum dots. Nano Lett. 2004; 4: 11–18. 28890669 10.1021/nl0347334 PMC5588688

[12] Gomes SA, Vieira CS, Almeida DB, Santos-Mallet JR, Menna-Barreto RF, Cesar CL, et al CdTe and CdSe quantum dots cytotoxicity: a comparative study on microorganisms. Sensors (Basel). 2011; 11: 11664–11678. 10.3390/s111211664 22247686 PMC3252003

[13] Ostermeier M. Engineering allosteric protein switches by domain insertion. Protein Eng Des Sel. 2005; 18: 359–364. 16043448 10.1093/protein/gzi048

[14] Smith WE, Brownell J, White CC, Afsharinejad Z, Tsai J, Hu X, et al In vitro toxicity assessment of amphiphillic polymer-coated CdSe/ZnS quantum dots in two human liver cell models. ACS Nano. 2012; 6: 9475–9484. 10.1021/nn302288r 23039050 PMC3671920

[15] Anas A, Akita H, Harashima H, Itoh T, Ishikawa M, Biju V. Photosensitized breakage and damage of DNA by CdSe-ZnS quantum dots. J Phys Chem B. 2008; 112: 10005–10011. 10.1021/jp8018606 18582008

[16] Hoshino A, Hanada S, Yamamoto K. Toxicity of nanocrystal quantum dots: the relevance of surface modifications. Arch Toxicol. 2011; 85: 707–720. 10.1007/s00204-011-0695-0 21445587

[17] Yong KT, Law WC, Hu R, Ye L, Liu L, Swihart MT, et al Nanotoxicity assessment of quantum dots: from cellular to primate studies. Chem Soc Rev. 2013; 42: 1236–1250. 10.1039/c2cs35392j 23175134

[18] Kloepfer JA, Mielke RE, Nadeau JL. Uptake of CdSe and CdSe/ZnS quantum dots into bacteria via purine-dependent mechanisms. Appl Env Microbiol. 2005; 71: 2548–2557. 15870345 10.1128/AEM.71.5.2548-2557.2005 PMC1087584

[19] Mahendra S, Zhu H, Colvin VL, Alvarez PJ. Quantum dot weathering results in microbial toxicity. Environ Sci Technol. 2008; 42: 9424–9430. 19174926 10.1021/es8023385

[20] Schneider R, Wolpert C, Guilloteau H, Balan L, Lambert J, Merlin C. The exposure of bacteria to CdTe-core quantum dots: the importance of surface chemistry on cytotoxicity. Nanotechnology. 2009; 20: 225101 10.1088/0957-4484/20/22/225101 19433881

[21] Zhou W, Baneyx F. Aqueous, protein-driven synthesis of transition metal-doped ZnS immuno-quantum dots. ACS Nano. 2011; 5: 8013–8018. 10.1021/nn2024896 21942544 PMC3204801

[22] Zhou W, Schwartz DT, Baneyx F. Single pot biofabrication of zinc sulfide immuno-quantum dots. J Am Chem Soc. 2010; 132: 4731–4738. 10.1021/ja909406n 20218715

[23] Zhou W, Swift BJF, Baneyx F. A minimized designer protein for facile biofabrication of ZnS:Mn immuno-quantum dots Chem Commun. 2015; Submitted. 10.1039/c4cc09531f PMC4326550 25571979

[24] Nies DH. Microbial heavy metal resistance. Appl Microbiol Biotechnol. 1999; 51: 730–750. 10422221 10.1007/s002530051457

[25] Silhavy TJ, Kahne D, Walker S. The bacterial cell envelope. Cold Spring Harb Perspect Biol. 2010; 2: a000414 10.1101/cshperspect.a000414 20452953 PMC2857177

[26] Nikaido H. Molecular basis of bacterial outer membrane permeability revisited. Microbiol Mol Biol Rev. 2003; 67: 593–656. 14665678 10.1128/MMBR.67.4.593-656.2003 PMC309051

[27] Wenhua L, Haiyan X, Zhixiong X, Zhexue L, Jianhong O, Xiangdong C, et al Exploring the mechanism of competence development in *Escherichia coli* using quantum dots as fluorescent probes. J Biochem Biophys Methods. 2004; 58: 59–66. 14597189

[28] Minsky A, Summers RG, Knowles JR. Secretion of beta-lactamase into the periplasm of *Escherichia coli*: evidence for a distinct release step associated with a conformational change. Proc Natl Acad Sci U S A. 1986; 83: 4180–4184. 3520569 10.1073/pnas.83.12.4180 PMC323695

[29] Shaner NC, Campbell RE, Steinbach PA, Giepmans BNG, Palmer AE, Tsien RY. Improved monomeric red, orange and yellow fluorescent proteins derived from *Discosoma* sp. red fluorescent protein. Nat Biotechnol. 2004; 22: 1567–1572. 15558047 10.1038/nbt1037

[30] Bayer ME. Areas of adhesion between wall and membrane of *Escherichia coli* . J Gen Microbiol. 1968; 53: 395–404. 4181162 10.1099/00221287-53-3-395

[31] Ruiz N, Kahne D, Silhavy TJ. Advances in understanding bacterial outer-membrane biogenesis. Nat Rev Microbiol. 2006; 4: 57–66. 16357861 10.1038/nrmicro1322

[32] Bianchi AA, Baneyx F. Stress responses as a tool to detect and characterize the mode of action of antibacterial agents. Appl Environ Microbiol. 1999; 65: 5023–5027. 10543818 10.1128/aem.65.11.5023-5027.1999 PMC91676

[33] Shapiro E, Baneyx F. Stress-based identification and classification of antibacterial agents: second generation Escherichia coli reporter strains and optimization of detection. Antimicrob Agents Chemother. 2002; 46: 2490–2497. 12121923 10.1128/AAC.46.8.2490-2497.2002 PMC127359

[34] Dumas EM, Ozenne V, Mielke RE, Nadeau JL. Toxicity of CdTe quantum dots in bacterial strains. IEEE Trans Nanobioscience. 2009; 8: 58–64. 10.1109/TNB.2009.2017313 19304497

[35] Dwarakanath S, Bruno JG, Athmaram TN, Bali G, Vattem D, Rao P. Antibody-quantum dot conjugates exhibit enhanced antibacterial effect vs. unconjugated quantum dots. Folia Microbiol. 2007; 52: 31–34. 17571792 10.1007/BF02932134

[36] Kloepfer JA, Mielke RE, Wong MS, Nealson KH, Stucky G, Nadeau JL. Quantum dots as strain- and metabolism-specific microbiological labels. Appl Env Microbiol. 2003; 69: 1205–4213. 10.1128/AEM.69.7.4205-4213.2003 PMC165133 12839801

[37] Lu Z, Li CM, Bao H, Qiao Y, Toh Y, Yang X. Mechanism of antimicrobial actvity of CdTe quantum dots. Langmuir. 2008; 24: 5445–5452. 10.1021/la704075r 18419147

[38] Imlay JA. Cellular defense against superoxide and hydrogen peroxide. Annu Rev Biochem. 2008; 77: 755–776. 10.1146/annurev.biochem.77.061606.161055 18173371 PMC3057177

[39] Koronakis V, Eswaran J, Hughes C. Structure and function of TolC: the bacterial exit duct for proteins and drugs. Annu Rev Biochem. 2004; 73: 467–489. 15189150 10.1146/annurev.biochem.73.011303.074104

[40] He Y, Wang H-F, Yan X-P. Exploring Mn-Doped ZnS Quantum Dots for the Room-Temperature Phosphorescence Detection of Enoxacin in Biological Fluids. Analytical Chemistry. 2008; 80: 3832–3837. 10.1021/ac800100y 18407673

[41] Silhavy TJB M. L.; Enquist L. W. Experiments with Gene Fusions. Cold Spring Harbor, NY: Cold Spring Harbor Laboratory Press 1984.

[42] Shapiro E, Baneyx F. Stress-Based Identification and Classification of Antibacterial Agents: Second-Generation Escherichia coli Reporter Strains and Optimization of Detection. Antimicrobial Agents and Chemotherapy. 2002; 46: 2490–2497. 12121923 10.1128/AAC.46.8.2490-2497.2002 PMC127359

