# Supplementary material for: Microbial Uptake, Toxicity, and Fate of Biofabricated ZnS:Mn Nanocrystals
Source: PLoS One. 2015 Apr 22;10(4):e0124916. doi: 10.1371/journal.pone.0124916 (PMC4406734; doi:10.1371/journal.pone.0124916)
Supplement: S1 Fig — Competent AB734 cells incubated with 0.5 μg/mL of BB-CT43-stabilized QDs remain fluorescent after 24h of incubation in PBS buffer at temperatures varying from 4 to 42°C. (PDF) [file pone.0124916.s001.pdf]

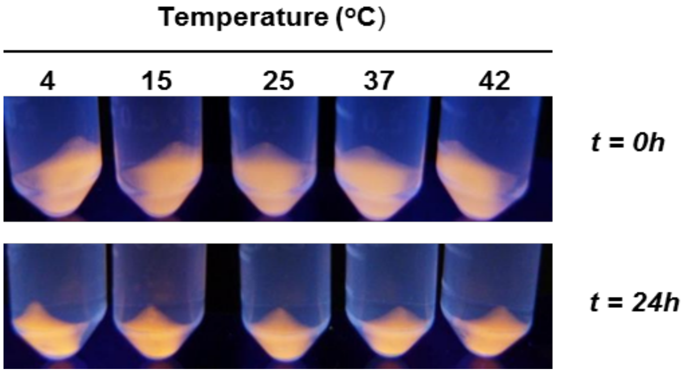

Figure S1. Quiescent cells that have internalized QDs remain fluorescent over a broad range of temperatures. Competent AB734 cells were incubated with 0.5 μg/mL of BB-CT43-stabilized nanocrystals, washed and centrifuged as described in the main text. Pellets were photographed under UV light (top panel). The cells were resuspended in 1 mL of PBS and incubated at the indicated temperatures for 24h, before being centrifuged and photographed as above.
