# Supplementary material for: Microbial Uptake, Toxicity, and Fate of Biofabricated ZnS:Mn Nanocrystals
Source: PLoS One. 2015 Apr 22;10(4):e0124916. doi: 10.1371/journal.pone.0124916 (PMC4406734; doi:10.1371/journal.pone.0124916)
Supplement: S2 Fig — Competent AB734 cells incubated with 0.5 μg/mL of BB-CT43-stabilized QDs remain fluorescent after 3h of incubation in PBS buffer at 37°C while they completely lose fluorescence under the same conditions in LB medium. (PDF) [file pone.0124916.s002.pdf]

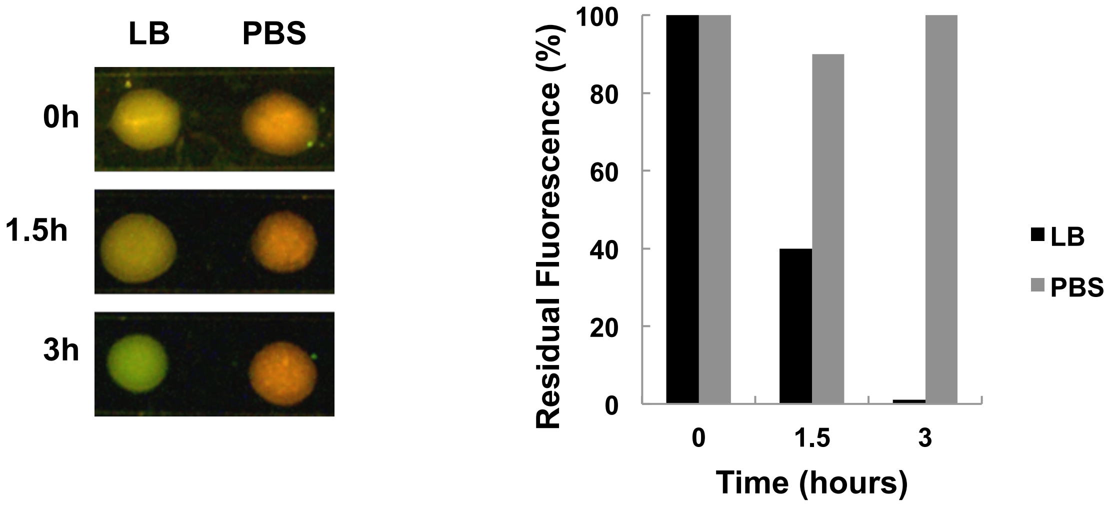

Figure S2. Quiescent cells that have internalized QDs remain fluorescent over time. AB734 cells were incubated with 0.5 μg/mL of BB-CT43-stabilized nanocrystals, washed and centrifuged as described in the main text. Cells were resuspended in 1 mL of LB medium or PBS. Photographs show the fluorescence of cell samples (50 μL) after the indicated incubation times at 37°C in LB or PBS. Fluorescence was quantified as described in Materials and Methods.
