# Supplementary material for: Microbial Uptake, Toxicity, and Fate of Biofabricated ZnS:Mn Nanocrystals
Source: PLoS One. 2015 Apr 22;10(4):e0124916. doi: 10.1371/journal.pone.0124916 (PMC4406734; doi:10.1371/journal.pone.0124916)
Supplement: S3 Fig — Inactivation of tolC has no obvious impact on the loss of fluorescence in AB734 cells experiencing balanced growth in LB medium at 37°C. (PDF) [file pone.0124916.s003.pdf]

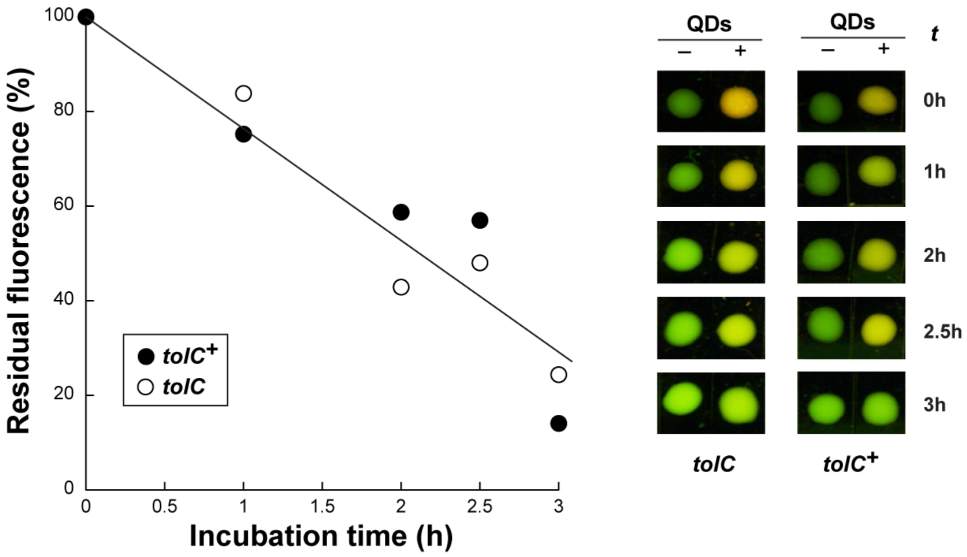

Figure S3. Inactivation of TolC does not impact fluorescence loss in metabolically active cells. The fluorescence of AB734 variants with an intact (closed circles) or disrupted (open circles) *tolC* gene[1] was quantified at the indicated time points following internalization of 0.5 μg/mL of BB-CT43-stabilized QDs and incubation at 37°C in LB medium. Photographs show the fluorescence of cell samples (50 μL) from cultures supplied (+) or not (-) with QDs after the indicated incubation times.

1. Shapiro, E. and F. Baneyx, *Stress-Based Identification and Classification of Antibacterial Agents: Second-Generation Escherichia coli Reporter Strains and Optimization of Detection*. Antimicrobial Agents and Chemotherapy, 2002. **46**(8): p. 2490-2497.
